# Characterization of three mycobacterial DinB (DNA polymerase IV) paralogs highlights DinB2 as naturally adept at ribonucleotide incorporation

**DOI:** 10.1093/nar/gku752

**Published:** 2014-09-08

**Authors:** Heather Ordonez, Maria Loressa Uson, Stewart Shuman

**Affiliations:** Molecular Biology Program, Sloan-Kettering Institute, New York, NY 10065, USA

## Abstract

This study unveils *Mycobacterium smegmatis* DinB2 as the founder of a clade of Y-family DNA polymerase that is naturally adept at incorporating ribonucleotides by virtue of a leucine in lieu of a canonical aromatic steric gate. DinB2 efficiently scavenges limiting dNTP and rNTP substrates in the presence of manganese. DinB2's sugar selectivity factor, gauged by rates of manganese-dependent dNMP versus rNMP addition, is 2.7- to 3.8-fold. DinB2 embeds ribonucleotides during DNA synthesis when rCTP and dCTP are at equimolar concentration. DinB2 can incorporate at least 16 consecutive ribonucleotides. In magnesium, DinB2 has a 26- to 78-fold lower affinity for rNTPs than dNTPs, but only a 2.6- to 6-fold differential in rates of deoxy versus ribo addition (*k*_pol_). Two other *M. smegmatis* Y-family polymerases, DinB1 and DinB3, are characterized here as template-dependent DNA polymerases that discriminate strongly against ribonucleotides, a property that, in the case of DinB1, correlates with its aromatic steric gate side chain. We speculate that the unique ability of DinB2 to utilize rNTPs might allow for DNA repair with a ‘ribo patch’ when dNTPs are limiting. Phylogenetic analysis reveals DinB2-like polymerases, with leucine, isoleucine or valine steric gates, in many taxa of the phylum *Actinobacteria.*

## INTRODUCTION

We are interested in the DNA repair strategies of the human pathogen *Mycobacterium tuberculosis* and its avirulent relative *M. smegmatis.* To this end, we are interrogating the large and distinctive roster of mycobacterial DNA ligases (1–3), DNA helicases (4–12) and DNA polymerases (13–15). The present study focuses on DNA polymerases, of which *M. smegmatis* encodes nine. Mizrahi *et al.* have previously characterized mycobacterial DNA polymerase I (PolA), DnaE1 and DnaE2 (16–20). DnaE1 is essential and is presumed to be the replicative polymerase. DnaE2, though inessential, is involved in adaptive mutagenesis and contributes to antibiotic resistance (19,20). Mycobacterial Pol1 differs from *Escherichia coli* Pol1 in that it lacks a proofreading 3′–5′ exonuclease activity, though it retains the 5′–3′ exonuclease activity (18), which is implicated in the 5′ processing of Okazaki fragments. A *polA* mutant of *M. smegmatis* in which the polymerase domain was disrupted, while preserving the 5′–3′ exonuclease, was viable but hypersensitive to UV and hydrogen peroxide (17).

DNA ligase D (LigD) is the central agent of the non-homologous end joining (NHEJ) pathway of bacterial DNA double strand break (DSB) repair (21). LigD is a modular enzyme composed of an ATP-dependent ligase domain, a 3′-phosphoesterase domain and a polymerase domain (LigD-POL). LigD-POL is proficient at adding templated and non-templated deoxynucleotides and ribonucleotides to DNA ends *in vitro*. In *M. smegmatis*, LigD-POL is the singular catalyst *in vivo* of mutagenic NHEJ events involving non-templated single-nucleotide additions to blunt DSB ends (13,14). By contrast, templated nucleotide insertions at 5′ overhang DSBs formed by restriction endonuclease cleavage were unaffected by inactivation of LigD-POL, signifying that *M. smegmatis* must have at least one other polymerase that fills in 5′ overhangs during NHEJ. Crystal structures of LigD-POL domains highlight its membership in the archaeal/eukaryal primase–polymerase (AEP) family (13,22,23). *M. smegmatis* and *M. tuberculosis* each encode two additional paralogous AEP proteins, PolD1 and PolD2, that are stand-alone homologs of the LigD-POL domain (15). Biochemical characterization of PolD1 and PolD2 showed that they resemble LigD POL in their ability to add templated and non-templated nucleotides to primer–templates and blunt ends, and their preference for rNTPs versus dNTPs (15). Deletion of *polD1*, *polD2* or both, in an *M. smegmatis* strain carrying an inactivating mutation in LigD-POL failed to reveal a role for PolD1 or PolD2 in templated nucleotide additions during NHEJ of 5′ overhang DSBs (15), thereby implying that yet other mycobacterial polymerases can perform this fill-in synthesis step.

In addition to the aforementioned six DNA polymerases, the *M. tuberculosis* proteome includes two homologs of the prototypal Y-family polymerase DinB (also known as DNA polymerase IV) (24). *Escherichia coli* DinB, the founding member of this family, is involved in adaptive mutagenesis (25–28) and in bypassing bulky adducts such as N^2^-furfuryl-dG and alkylated purines 3-methyl-dA and 3-methyl-dG (29,30). *Escherichia coli dinB* is under the transcriptional control of the LexA repressor and is induced in response to DNA damage (31). The Mizrahi lab has conducted an analysis of the *M. tuberculosis* DinB1 and DinB2 paralogs that highlights stark differences in their regulation and biology vis à vis*E. coli* DinB (24). Specifically, they showed that (i) *dinB1* and *dinB2* are expressed constitutively during logarithmic growth and stationary phase; (ii) *dinB1* and *dinB2* are not induced by DNA damage or dependent on RecA and SOS-response; (iii) the steady-state RNA level for *dinB2* is 12-fold higher than *dinB1*; and (iv) DinB1 interacts in a yeast 2-hybrid assay with the mycobacterial β-clamp DNA polymerase processivity factor, whereas DinB2 does not (24). Their genetic analysis showed that (i) *M. tuberculosis dinB1*Δ, *dinB2*Δ and *dinB1*Δ *dinB2*Δ knockout strains grow normally in culture; (ii) *dinB* deletions have no effect on sensitivity to nitrofurazone, nitroquinolone oxide, ethyl methane sulfonate (EMS), methyl methane sulfonate (MMS), mitomycin C or novobiocin; (iii) there was no effect of *dinB* deletions on the rate of spontaneous mutation to rifampin-resistance; and (iv) *dinB* deletions had no effect on growth of *M. tuberculosis* in macrophages or mice (24).

The available evidence that DinB homologs from mycobacteria do not behave like their counterparts from other model organisms raises interesting questions about their biochemical activities and specificities. In the present study, we purify and characterize the *M. smegmatis* DinB1 and DinB2 paralogs, and we identify and characterize a third DinB3 paralog unique to *M. smegmatis*. Whereas DinB1 and DinB3 are typical DNA-dependent DNA polymerases, we report that DinB2 is uniquely and naturally adept at adding ribonucleotides during templated primer extension. We present a detailed analysis of DinB2 dNTP/rNTP substrate discrimination and pinpoint a crucial amino acid polymorphism at the steric gate that governs this parameter.

## MATERIALS and METHODS

### Cloning

The open reading frames (ORFs) encoding full-length *M. smegmatis* DinB1 (MSMEG_3172), DinB2 (MSMEG_2294), and DinB3 (MSMEG_6443) were PCR-amplified from *M. smegmatis* genomic DNA with primers that introduced a BglII site immediately flanking the start codon and a HindIII (DinB1) or XhoI (DinB2, DinB3) site downstream of the respective stop codons. The polymerase chain reaction (PCR) products were digested with BglII and HindIII for DinB1, and BglII and XhoI for DinB2 and DinB3, and ligated into pET28b-His_10_Smt3 that had been digested with BamHI and HindIII or XhoI. The resulting pET28b-His_10_Smt3-DinB1, pET28b-His_10_Smt3-DinB2 or pET28b-His_10_Smt3-DinB3 expression plasmids encode DinB1, DinB2 and DinB3 polypeptides fused to an N-terminal His_10_Smt3 tag under the transcriptional control of a T7 RNA polymerase promoter. Plasmids encoding mutants DinB1-(D113A), DinB1-(F23A), DinB1-(F23L), DinB2-(D107A), DinB2-(L14A) and DinB2-(L14F) were obtained by site-directed mutagenesis using the quick change method with NEB Phusion polymerase. The plasmid inserts were sequenced to verify that no unintended coding changes were acquired during amplification and cloning.

### Recombinant DinB

The pET28b-His_10_Smt3-DinB1, pET28b-His_10_Smt3-DinB2 and pET28b-His_10_Smt3-DinB3 plasmids were transformed into *E. coli* BL21(DE3) cells. Cultures (1 liter) amplified from single kanamycin-resistant transformants were grown at 37°C in Luria-Bertani (LB) broth containing 60 μg/ml kanamycin until the *A*_600_ reached 0.5. The cultures were chilled on ice for 1 h, then adjusted to 2% (v/v) ethanol and 0.4 mM isopropyl-β-D-thiogalactopyranoside and incubated for 16–18 h at 17°C with constant shaking. All subsequent steps were performed at 4°C. Cells were harvested by centrifugation and resuspended in 25 ml of buffer A (50 mM Tris-HCl, pH 8.0, 500 mM NaCl, 1 mM DTT, 10% sucrose, 20 mM imidizole) containing 1 protease inhibitor cocktail tablet (Roche). Lysozyme was added to a concentration of 1 mg/ml. After incubation for 1 h, the lysate was sonicated to reduce viscosity and the insoluble material was pelleted by centrifugation at 38 000 g for 35 min. The supernatant was mixed for 1 h with 3 ml of Ni-NTA agarose resin (Qiagen) that had been equilibrated with buffer A. The resin was recovered by centrifugation and resuspended in 30 ml of buffer A. The washed resin was then recovered by centrifugation, resuspended in 10 ml of buffer A and poured into a column. The column was washed serially with 4 column volumes of 3 M KCl, 4 column volumes of buffer B (50 mM Tris-HCl, pH 8.0, 500 mM NaCl, 10% glycerol) containing 20 mM imidazole, and 2 column volumes of 50 mM imidazole in buffer B. The bound protein was eluted with 500 mM imidizole in buffer B. The polypeptide compositions of the fractions were monitored by sodium dodecylsulphate-polyacrylamide gel electrophoresis (SDS-PAGE). The 500 mM imidazole eluates containing His_10_Smt3-DinB were supplemented with 10 mM ethylenediaminetetraacetic acid (EDTA) and the Smt3-specific protease Ulp1 (60 μg) and then dialyzed against 4 l of buffer C (50 mM Tris-HCl, pH 8.0, 500 mM NaCl, 1 mM DTT, 20 mM imidazole, 10% glycerol) for 16 h, during which time the His_10_Smt3 tag was cleaved. The dialysates were mixed with 3 ml of Ni-NTA agarose equilibrated with buffer C. The mixtures were nutated for 1 h and then poured into a column; the tag-free DinB1, DinB2 and DinB3 proteins were recovered in the flow-through fractions. The DinB1 and DinB2 protein preparations were purified further by gel filtration through a Superdex-200 column equilibrated in buffer D (500 mM NaCl, 10% glycerol, 10 mM Tris-HCl, pH 8.0, 1 mM DTT, 1 mM EDTA). Peak DinB1 and DinB2 fractions were pooled and stored at **−**80°C. Protein concentrations were determined with the BioRad dye reagent using BSA as the standard. The yields of DinB1 and DinB2 and corresponding mutants were ∼15 mg per liter of culture. The yield for DinB3 was ∼3 mg per liter of culture.

### Primer–templates

The 5′ ^32^P-labeled primer DNA strand was prepared by reaction of a synthetic oligodeoxynucleotide with T4 polynucleotide kinase and [γ^32^P]ATP. The labeled DNA was heated to 95°C to inactivate the kinase. Primer DNA was then annealed to a 3-fold excess of a complementary DNA strand to form the primer–templates shown in the figures. The annealed DNAs were purified by electrophoresis through a native 15% polyacrylamide gel and then eluted from an excised gel slice by incubation for 16 h at 4°C in 300 μl of 10 mM Tris-HCl, pH 7.5, 1 mM EDTA, 50 mM NaCl.

### Polymerase assay

Reaction mixtures containing 10 mM Tris-HCl, pH 7.5, 50 or 100 nM 5′ ^32^P-labeled primer–template, and divalent cations, dNTP or rNTPs, and DinB enzyme as specified in the figure legends were incubated at 37°C. The reactions were initiated by adding DinB and quenched with an equal volume of 90% formamide, 50 mM EDTA and 0.3% bromophenol blue. The reaction products were analyzed by electrophoresis through either a 40 cm or 15 cm 18% polyacrylamide gel containing 7 M urea in 89 mM Tris-borate, 2.5 mM EDTA. The products were visualized by autoradiography and, where indicated, quantified by scanning with a Fujix imager.

## RESULTS

### Mycobacterial DinB1 and DinB2 are DNA polymerases

**Figure 1.**
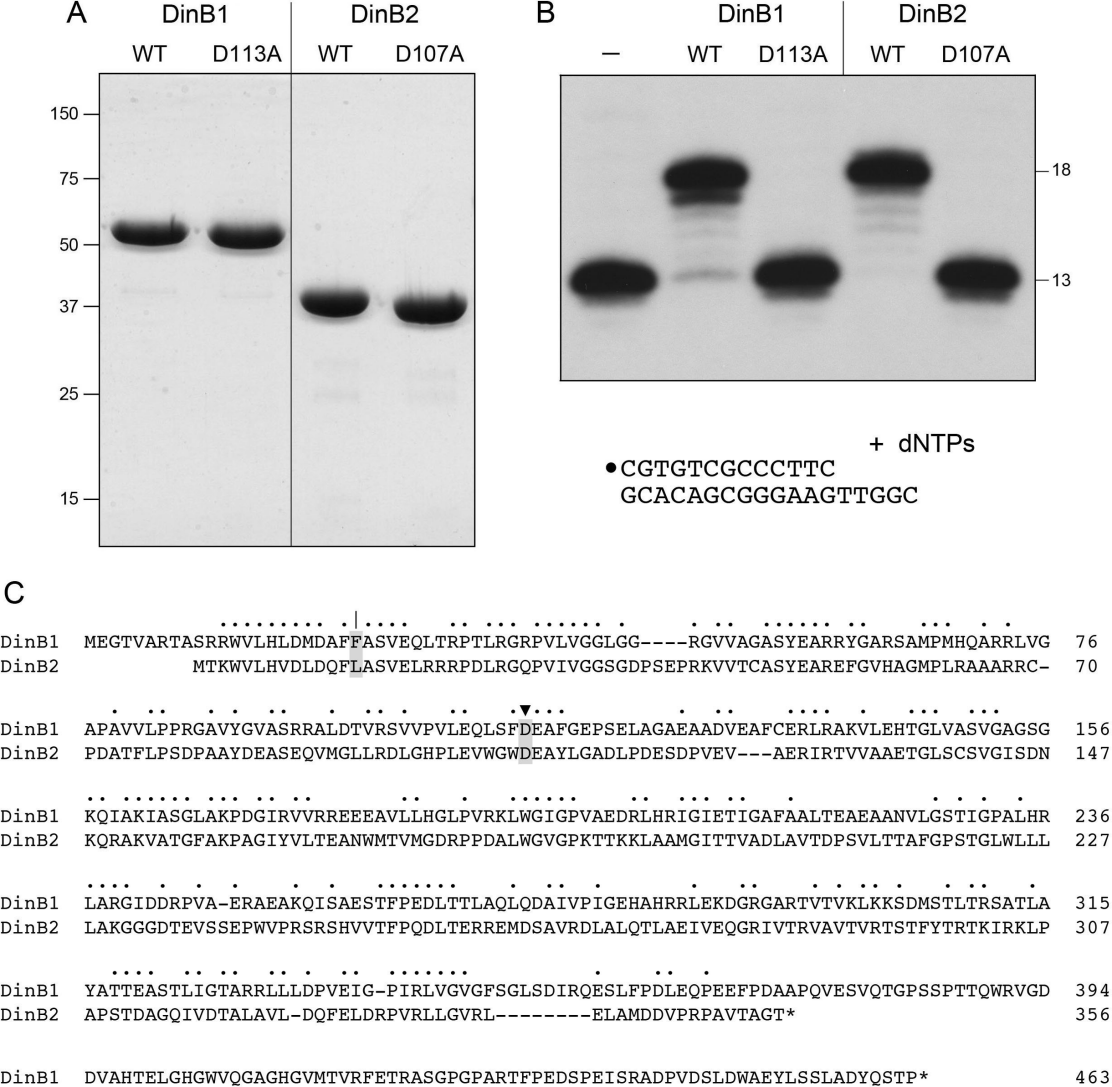
DinB1 and DinB2 are DNA polymerases. (**A**) Purification. Aliquots (5 μg) of recombinant wild-type DinB1, DinB1-D113A, wild-type DinB2 and DinB2-D107A were analyzed by SDS-PAGE. The Coomassie Blue-stained gel is shown. The positions and sizes (kDa) of marker polypeptides are indicated on the left. (**B**) DNA polymerase reaction mixtures (10 μl) containing 10 mM Tris-HCl, pH 7.5, 5 mM MnCl_2_, 125 μM each of dATP, dGTP, dCTP and dTTP (dNTPs), 100 nM 5′ ^32^P-labeled 13-mer/18-mer primer–template (depicted at bottom, with the 5′ ^32^P label denoted by •) and 500 nM of the DinB protein were incubated at 37°C for 15 min. The reaction products were analyzed by urea-PAGE and visualized by autoradiography. (**C**) DinB1 and DinB2 primary structures. The amino acid sequences of the MsmDinB1 and MsmDinB2 proteins are aligned. Positions of side chain identity/similarity are denoted by dots. Gaps in the alignments are denoted by dashes. The metal-binding aspartate residues Asp113 and Asp107 that were mutated in (A) and (B) are shaded and denoted by ▾. The steric gate residues are shaded and denoted by |.

*Mycobacterium tuberculosis* encodes two DinB paralogs: MtuDinB1 (Rv1537) and MtuDinB2 (Rv3056). The equivalent *M. smegmatis* proteins are MsmDinB1 (MSMEG_3172) and MsmDinB2 (MSMEG_2294). Alignment of the 463-aa MsmDinB1 and 356-aa MsmDinB2 polypeptides highlights 123 positions of amino acid identity and 43 positions of side-chain similarity (Figure 1C). In order to characterize the enzymatic activities of MsmDinB1 and MsmDinB2, we produced them in *E. coli* as His_10_Smt3 fusions and isolated the recombinant proteins from soluble cell extracts by nickel-agarose chromatography. The His_10_Smt3 tag was removed with the Smt3-specific protease Ulp1, and native DinB1 and DinB2 proteins were separated from the tag by a second round of Ni-agarose chromatography. The tag-free DinBs were further purified by gel filtration, during which DinB1 and DinB2 eluted as single discrete peaks at elution volumes consistent with each being a monomer in solution (not shown). SDS-PAGE verified the purity of the 50 kDa and 39 kDa polypeptides corresponding to DinB1 and DinB2, respectively (Figure 1A). DinB1 and DinB2 catalyzed templated DNA synthesis, as gauged by their ability, in the presence of a divalent cation and the four dNTPs (125 μM each), to extend a 5′ ^32^P-labeled 13-mer DNA primer strand annealed to a complementary 18-mer template strand (Figure 1B).

*Escherichia coli* DinB employs a two-metal mechanism of nucleotide addition to the 3′-OH primer terminus (32). The metals are coordinated by three carboxylate side chains (Asp8, Asp103, Glu104) that are conserved in *M. smegmatis* DinB1 (Asp18, Asp113, Glu114) and DinB2 (Asp9, Asp107, Glu108). We produced and purified mutated versions of DinB1 and DinB2 in which the metal-binding Asp113 and Asp107 residues (denoted by ▾ in Figure 1C) were replaced by alanine (Figure 1A). These mutations abolished the DNA polymerase activity of DinB1 and DinB2 (Figure 1B). We conclude that the polymerase activities are intrinsic to the recombinant DinB1 and DinB2 proteins.

### Divalent cation specificities of DinB1 and DinB2 DNA polymerases

**Figure 2.**
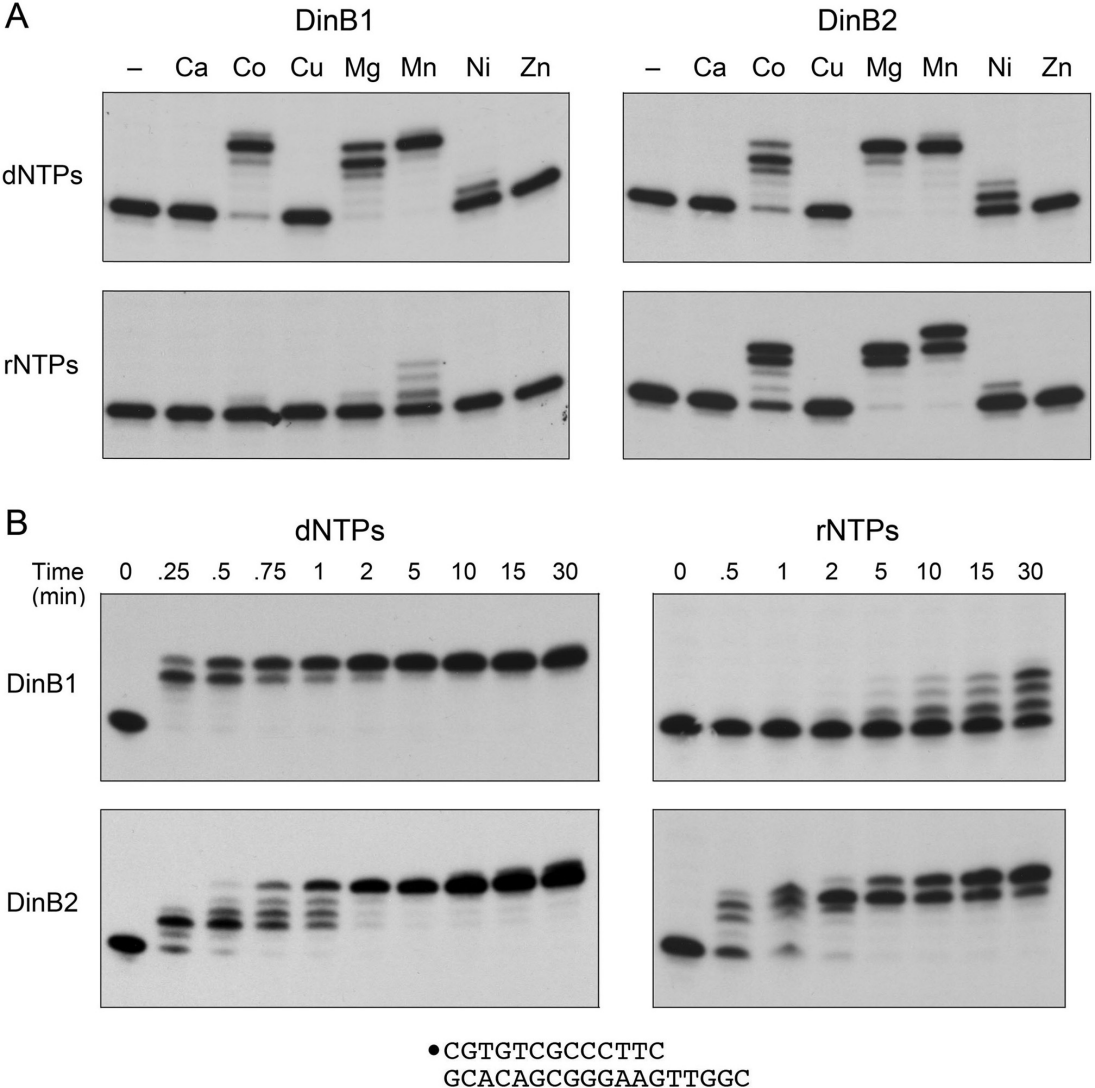
Characterization of DinB1 and DinB2 polymerase activities. (**A**) Divalent cation specificity. Polymerase reaction mixtures (10 μl) containing 10 mM Tris-HCl, pH 7.5, 100 nM 5′ ^32^P-labeled 13-mer/18-mer primer–template, 125 μM each of dATP, dGTP, dCTP and dTTP (dNTPs, top panels) or ATP, GTP, CTP and UTP (rNTPs, bottom panels) as specified, 1 μM DinB1 (left panels) or DinB2 (right panels) as indicated, and either no added metal cofactor (lanes –) or 5 mM of the indicated divalent cation (as the chloride salt) were incubated at 37°C for 15 min. (**B**) Kinetic profiles. Polymerase reaction mixtures containing 10 mM Tris-HCl, pH 7.5, 5 mM MnCl_2_, 100 nM 5′ ^32^P-labeled 13-mer/18-mer primer–template, 125 μM dNTP (left panels) or rNTPs (right panels), and 1 μM DinB1 (top panels) or DinB2 (bottom panels) were incubated at 37°C. Aliquots (10 μl) were withdrawn at the times specified and quenched immediately with EDTA/formamide. The reaction products were analyzed by urea-PAGE and visualized by autoradiography.

DNA synthesis by DinB1 and DinB2 was strictly dependent on provision of exogenous divalent cation (Figure 2A; dNTPs, lanes –). Comparing various metals at 5 mM (as the chloride salt), we found that activity was optimal with manganese, with respect to primer utilization (the percent of input primer extended by at least one dNMP addition after 15 min reaction in enzyme excess) and the completeness of synthesis to the end of the template strand (Figure 2A). In manganese, DinB1 and DinB2 performed five cycles of dNTP addition to achieve complete fill-in of all the available primer. Although magnesium supported efficient primer utilization by both enzymes, the DinB1 reaction generated a mixture of products extended by four or five dNMP steps, with the +4 additions predominating, indicative of either pausing or dissociation of the polymerase prior to the last templated addition. Cobalt supported complete fill-in DNA synthesis by DinB1, but it caused DinB2 to accumulate the +4 extension product. Nickel was a feeble cofactor for DinB1 and DinB2; neither DNA polymerase was active in the presence of calcium, copper or zinc (Figure 2A).

The kinetic profiles of manganese-dependent dNMP addition by DinB1 and DinB2 are shown in Figure 2B (dNTPs). DinB1 extended all of the input primer by four nucleotides within 15 s, which was followed by a slow phase, from 15 s to 2 min, during which the final template dNMP was added to achieve complete fill-in (Figure 2B). By contrast, DinB2 paused after the incorporation of the first two dNMPs (which was complete within 15–30 s) before completing fill-in synthesis by 2–5 min. At longer reactions times (15 and 30 min) DinB2 generated low levels of a +6 extension product, which we presume reflects a single non-templated dNMP addition at the filled-in blunt duplex end (Figure 2B).

### DNA polymerase activity of *M. smegmatis* DinB3

**Figure 3.**
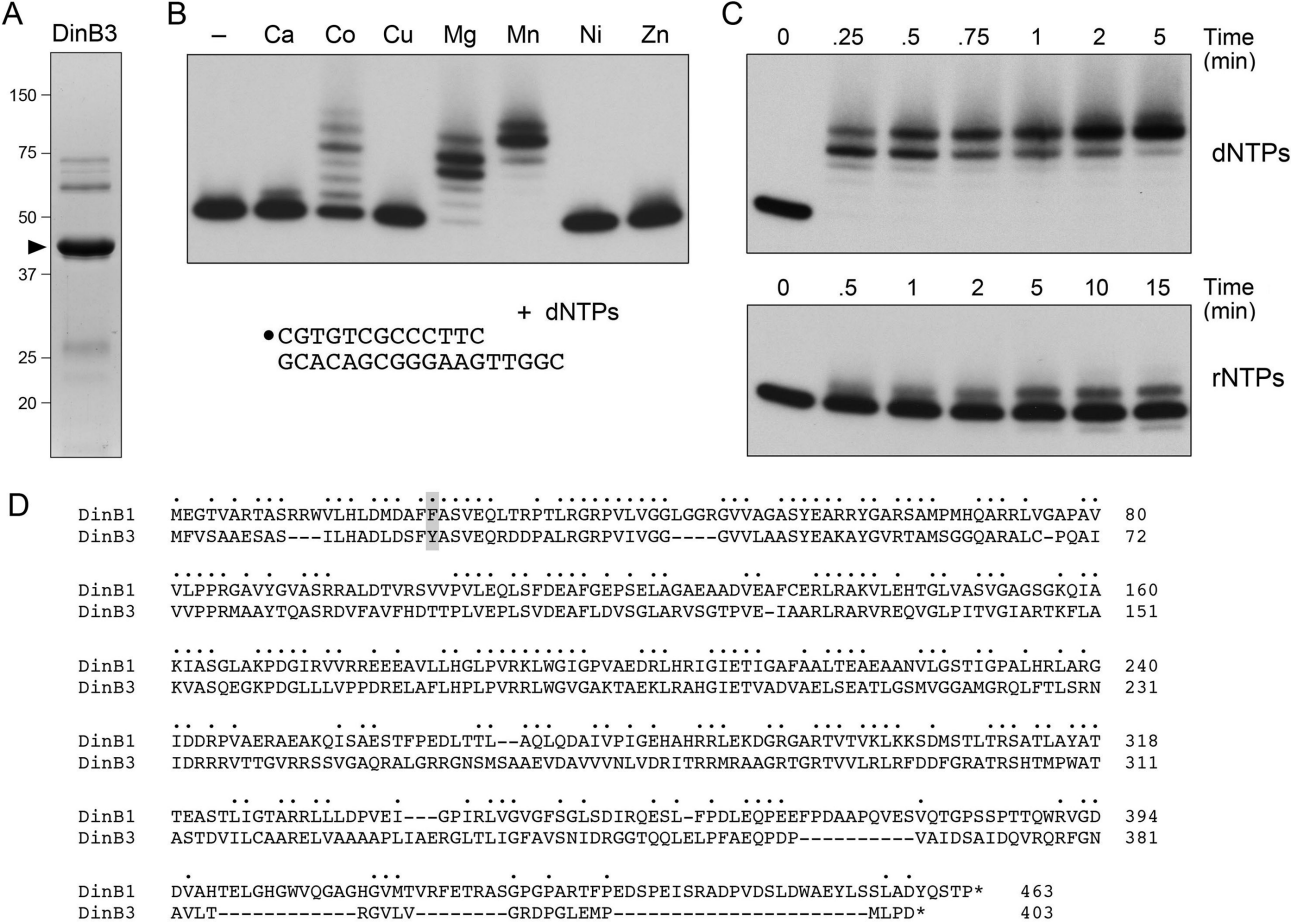
Characterization of DinB3 polymerase activity. (**A**) Purification. An aliquot (5 μg) of recombinant DinB3 was analyzed by SDS-PAGE. The Coomassie Blue-stained gel is shown. The positions and sizes (kDa) of marker polypeptides are indicated on the left. The DinB3 polypeptide is denoted by ▸. (**B**) Metal specificity. Polymerase reaction mixtures (10 μl) containing 10 mM Tris-HCl, pH 7.5, 100 nM 5′ ^32^P-labeled 13-mer/18-mer primer–template, 125 μM each of dATP, dGTP, dCTP and dTTP (dNTPs), 1 μM DinB3, and either no added metal cofactor (lane –) or 5 mM of the indicated divalent cation (as the chloride salt) were incubated at 37°C for 15 min. (**C**) Kinetic profile. Polymerase reaction mixtures containing 10 mM Tris-HCl, pH 7.5, 5 mM MnCl_2_, 100 nM 5′ ^32^P-labeled 13-mer/18-mer primer–template, 125 μM dNTPs (top panel) or rNTPs (bottom panel) and 1 μM DinB3 were incubated at 37°C. Aliquots (10 μl) were withdrawn at the times specified and quenched immediately with EDTA/formamide. The reaction products were analyzed by urea-PAGE and visualized by autoradiography. (**D**) DinB3 primary structure. The amino acid sequences of the MsmDinB1 and MsmDinB3 proteins are aligned. Positions of side chain identity/similarity are denoted by dots. Gaps in the alignments are denoted by dashes. The steric gate residues are shaded.

*Mycobacterium smegmatis* encodes a third DinB paralog (MSMEG_6443; hereafter named DinB3) that is not present in the proteome of *M. tuberculosis*. Alignment of the 403-aa DinB3 polypeptide to DinB1 highlights 153 positions of amino acid identity and 54 positions of side-chain similarity (Figure 3D). Thus, DinB1 and DinB3 are more closely related to each other than they are to DinB2. We produced DinB3 in *E. coli* and purified the recombinant protein via the same procedures used for DinB1 and DinB2 through the second Ni-affinity step; we thereby obtained a protein preparation in which the 43 kDa DinB3 polypeptide was the major constituent (Figure 3A). DinB3 catalyzed manganese-dependent addition of dNMPs to the 13-mer/18-mer primer–template that yielded a mixture of +5 (complete fill-in) and +6 (extra non-templated addition) 5′ ^32^P-labeled primer extension products (Figure 3B). In the presence of magnesium, DinB3 extended the primer by three or four steps of dNMP addition (Figure 3B). Cobalt was a feeble cofactor for the DinB3 DNA polymerase; calcium, copper, nickel and zinc were inactive (Figure 3B). The kinetic profile of fill-in synthesis by DinB3 (Figure 3C) was similar to that of DinB1. To wit, DinB3 elongated virtually all of the input primer by at least four nucleotides within 15 s, after which the final template dNMP was slowly added to attain complete fill-in at 5 min (Figure 3C; dNTPs). A minority of the ^32^P-labeled fill-in products underwent an extra step of non-templated addition (Figure 3B, Mn; Figure 3C, 5 min).

### DinB2 is uniquely adept at ribonucleotide addition to a DNA primer–template

Three of the mycobacterial DNA polymerases characterized previously—LigD-POL, PolD1 and PolD2—share a distinctive property of readily adding ribonucleotides to a DNA primer–template. A role for such an RNA polymerase activity during NHEJ and/or gap repair has been discussed as a potentially useful strategy for damage control in non-replicating bacteria that have limited pools of dNTPs versus rNTPs (21). Here we surveyed and compared the capacity of the three mycobacterial DinB paralogs to utilize rNTPs (125 μM each) as substrates in the DNA primer extension reaction.

DinB2 was uniquely adept as an RNA polymerase. Under the same primer–template, enzyme, and divalent cation concentrations used to assay DNA polymerase activity, DinB2 displayed a vigorous ribonucleotide incorporation activity that was sustained by manganese, magnesium or cobalt (Figure 2A; rNTPs). Manganese was the optimal cofactor with respect to the length of the primer extension products, which comprised a mixture of +4 and +5 species. Magnesium and cobalt yielded a mixture of +3 and +4 species (Figure 2A). Calcium, copper, nickel and zinc were ineffective. The kinetic profile of manganese-dependent rNMP addition by DinB2 is shown in Figure 2B. The rate of the first ribonucleotide addition step, gauged by the percent conversion of the input primer to all longer species (77% after 1 min), was about one-fourth that of the first dNMP addition (72% after 15 s) (Figure 2B, DinB2). A distinctive feature of the RNA fill-in reaction was a clear kinetic delay after the addition of four rNTPs (complete by 2–5 min) prior to the addition of a fifth ribonucleotide at the last template position, which proceeded slowly between 2 and 30 min (Figure 2B). A pause at +4 was not evident during dNMP addition by DinB2.

DinB1, by contrast, was quite feeble at ribonucleotide addition; in the presence of its favored metal manganese and sufficient DinB1 to effect complete fill-in DNA synthesis, DinB1 extended 35% of the input primer and did so by just one, two or three rNMP addition steps (Figure 2A). DinB1 was inactive as an RNA polymerase with any of the other metal cofactors tested (Figure 2A). The kinetic profile of manganese-dependent rNMP addition by DinB1 is shown in Figure 2B. The initial rNMP addition step was extremely slow (59% primer utilization in 30 min) compared to dNMP addition (>90% in 15 s). DinB3 was similarly feeble at ribonucleotide addition, which was inefficient with respect to primer utilization and limited to a single step of rNMP incorporation (Figure 3C).

### Longer tracts of templated synthesis by DinB1 and DinB2

**Figure 4.**
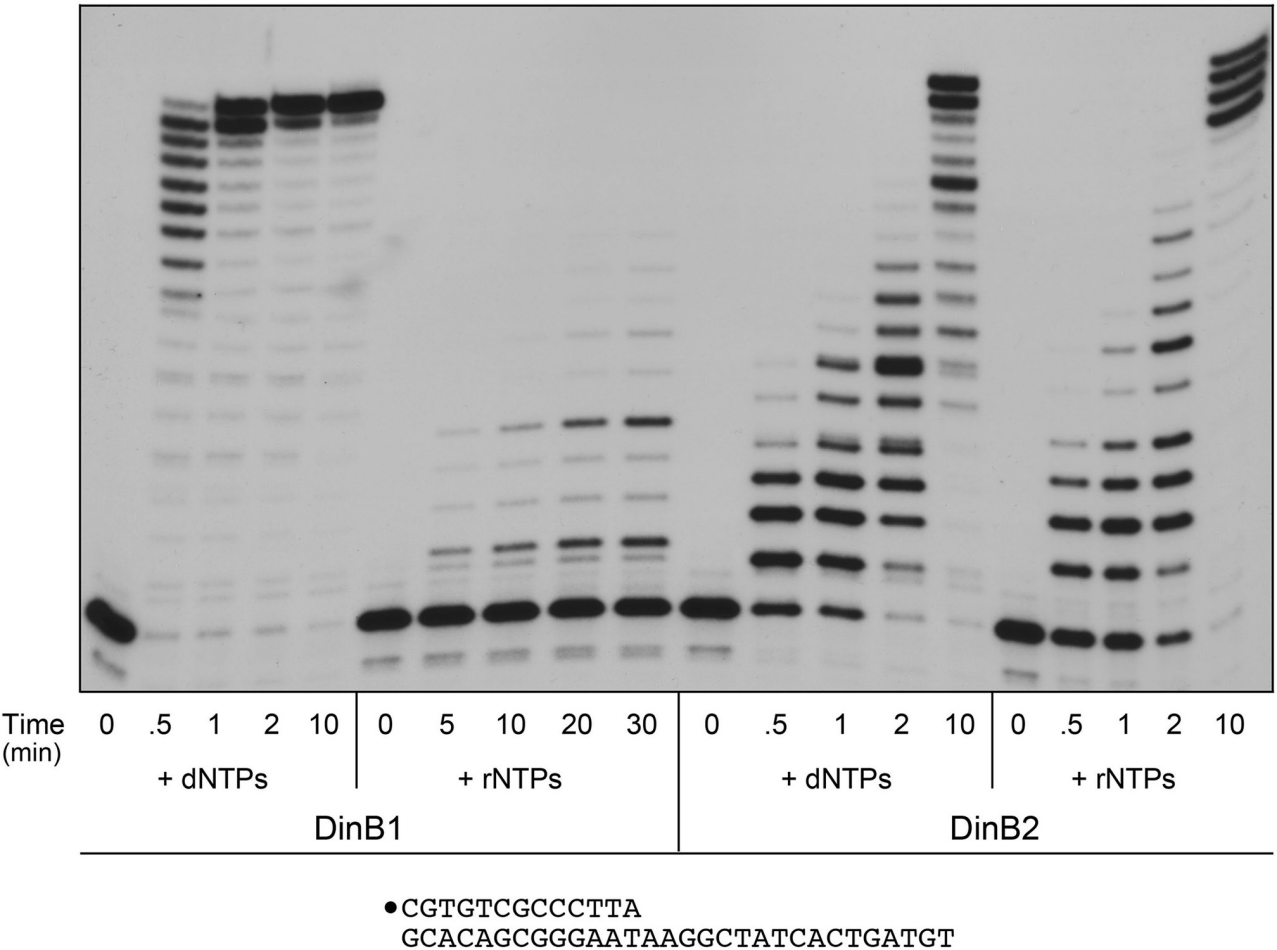
Longer tracts of templated synthesis by DinB1 and DinB2. Polymerase reaction mixtures containing 10 mM Tris-HCl, pH 7.5, 5 mM MnCl_2_, 50 nM 5′ ^32^P-labeled 13-mer/30-mer primer–template (depicted at bottom, with the 5′ ^32^P label denoted by •), dNTPs (125 μM each) or rNTPs (125 μM each) as specified, and 1 μM DinB1 or DinB2 were incubated at 37°C. Aliquots (10 μl) were withdrawn at the times specified and quenched immediately with EDTA/formamide. The reaction products were analyzed by urea-PAGE and visualized by autoradiography.

The primer extension product distributions observed with the 13-mer/18-mer primer–template suggested potentially interesting effects on the last step of fill-in synthesis, but we could not distinguish whether the seemingly slow last step reflects sensing of the number of addition steps performed or the proximity of the polymerase to the end of the template strand. To address this issue for DinB1, we employed a 5′ ^32^P-labeled primer–template comprising a 13-bp duplex with a 17-nucleotide 5′-tail to gauge the kinetics of manganese-dependent nucleotide addition (Figure 4). DinB1 extended virtually all of the input primer within 30 s in the presence of dNTPs, generating a cluster of products elongated by 10–16 steps of dNMP addition, but little in the way of the completely filled-in +17 product. From the leading edge, we can estimate that DinB1 incorporates ∼0.5 dNMP s^−1^. After 1 min, the DinB1 products comprised a roughly equal mixture of +16 and +17 species. The last templated addition step proceeded slowly between 1 and 10 min. We surmise that DinB1 experiences a kinetic delay when synthesis proceeds to one nucleotide from the template strand 5′ end, conceivably because the polymerase relies on contacts to the template single strand in advance of the primer 3′-OH terminus. We considered the possibility that the comparative weakness of DinB1 as an RNA polymerase might reflect a need for more extensive template strand contacts that were not satisfied by the 13-mer/18-mer primer–template. However, this notion was vitiated by the finding that DinB1 remained feeble in its ability to use rNTPs to fill in the longer template strand of the 13-mer/30-mer primer–template (Figure 4). The fraction of primers extended with ribonucleotides was low (29%) and the number of ribonucleotides added per primer was limited (mostly 1–4 steps) after a 30 min reaction with DinB1 (Figure 4), signifying an inherent bias in DinB1 against rNTPs as substrates, independent of template length.

The kinetic profile of DinB2 for dNMP addition to the 13-mer/30-mer primer–template revealed elongation by 1–3 nucleotides within 30 s, a pattern that persisted to 1 min, before further extension ensued at 2 min, culminating in the synthesis of +16 and +17 extension products at 10 min (Figure 4). The transient pause after a couple of dNMP additions by DinB2 was common to the 13-mer/18-mer and 13-mer/30-mer primer–templates, and thus not dictated by the length of the single-strand tail.

Previous studies had shown that bacterial LigD-POL, which favors rNTPs versus dNTPs as substrates for primer extension (33), can incorporate up to four sequential rNMPs at a DNA primer terminus, after which it ceases to elongate; this effect is attributed to the inability of LigD-POL to extend an RNA:DNA hybrid primer terminus with fully A-form helical conformation (34). Here we found that DinB2's RNA polymerase activity is not limited by ribonucleotide tract length, insofar as DinB2 catalyzed effective fill-in RNA synthesis on the 13-mer/30-mer primer–template (Figure 4). The kinetic profile for rNMP addition was quite similar to that of DNA synthesis, including the transient pause after extension of the primer by 1–3 nucleotides. By 10 min, DinB2 elongated virtually all of the input primer to form a cluster of +13, +14, +15 and +16 products (Figure 4).

### DinB2 can embed ribonucleotides during DNA synthesis

**Figure 5.**
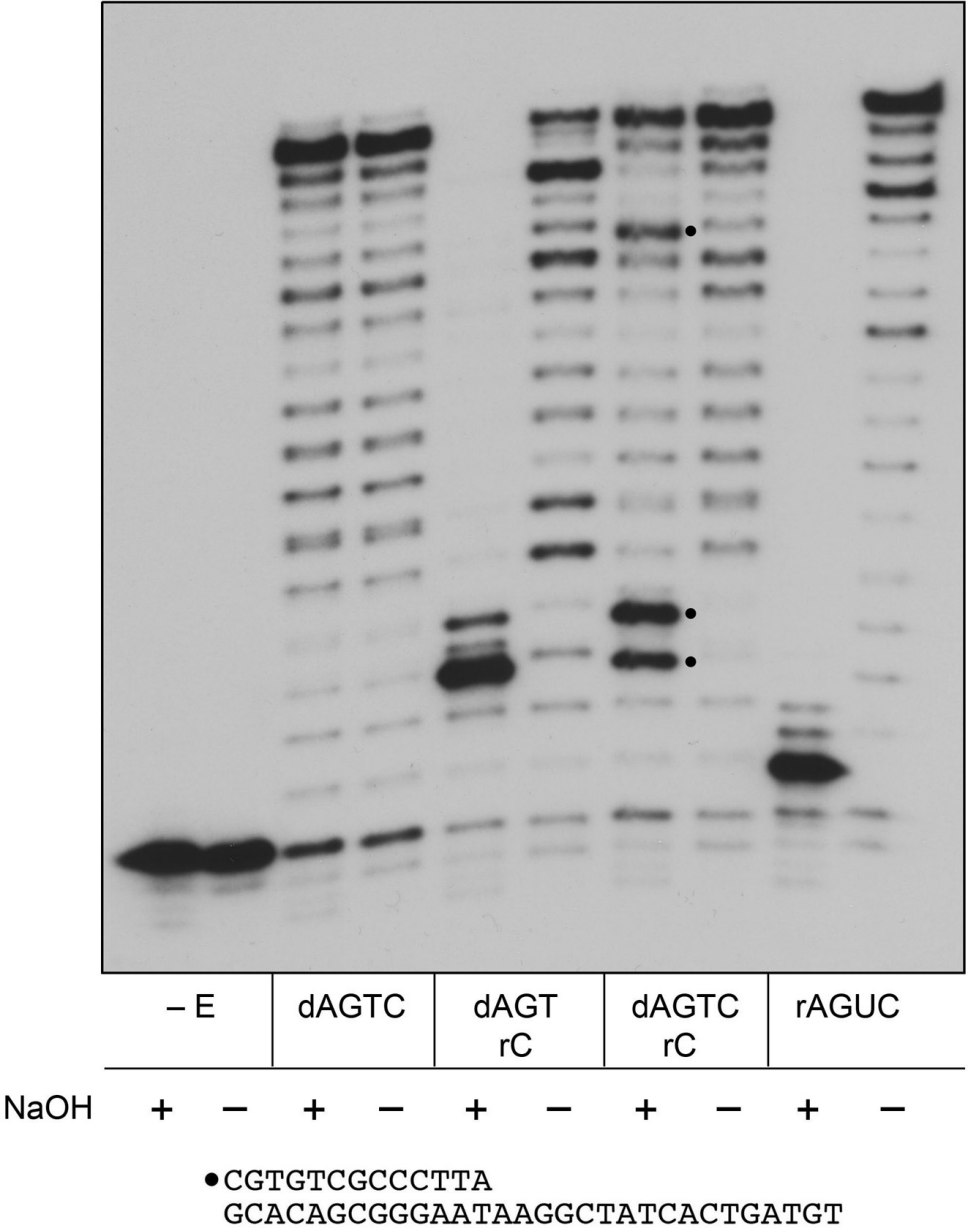
DinB2 can embed ribonucleotides during DNA synthesis. Polymerase reaction mixtures (20 μl) containing 10 mM Tris-HCl, pH 7.5, 5 mM MnCl_2_, 50 nM 5′ ^32^P-labeled 13-mer/30-mer primer–template, either 125 μM each of dATP, dGTP, dTTP and dCTP (dAGTC), 125 μM each of ATP, GTP, UTP and CTP (rAGUC), 125 μM each of dATP, dGTP, dTTP and rCTP (dAGT/rC), or 125 μM each of dATP, dGTP, dTTP plus 63 μM each of dCTP and rCTP (dAGTC/rC), and either 1 μM DinB2 or no enzyme (–E) were incubated for 20 min at 37°C. The reactions were quenched by adjustment to 20 mM EDTA. Aliquots (10 μl) of each sample were then adjusted to either 0.25 M NaOH (denoted by +) or 0.25 M NaCl (denoted by −) and incubated at 25°C for 16 h. The NaOH samples were then neutralized with 0.25 M HCl. The reaction products were analyzed by urea-PAGE and visualized by autoradiography. The three alkaline hydrolysis products indicative of rCMP incorporation at +3, +4 and +13 during DNA synthesis in the presence of all four dNTPs are indicated by •. The gel was scanned with a Fujix BAS2500 imager and the extents of rCMP incorporation at +3, +4 and +13 sites were calculated as described in the Results section.

To formally prove that DinB2 polymerizes an RNA tract on the 13-mer/30-mer primer–template, we gauged the sensitivity of the primer extension products to alkaline hydrolysis. The extended products made by DinB2 in the presence of dNTPs only were unaffected by overnight incubation in 0.25 M NaOH (Figure 5; dAGCT). By contrast, all the products synthesized by DinB2 in the presence of rNTPs were converted by NaOH treatment to a ^32^P-labeled species migrating about one nucleotide slower than the input 13-mer pDNA_OH_ primer (Figure 5; rAGUC), signifying that all of the newly synthesized polynucleotide tract consisted of ribonucleoside-3′-phosphodiesters. The ^32^P-labeled end product of alkaline hydrolysis comprised a mixture of pDNAp(rN)_2′_p and pDNAp(rN)_3′_p strands.

The role of the template nucleobases in directing ribonucleotide incorporation was established by reacting DinB2 with the 13-mer/30-mer primer–template in the presence of three dNTPs (dATP, dGTP, dTTP) and one rNTP (CTP). The template strand contains four guanine nucleobases that should, in principle, direct the inclusion of four rCMP nucleotides in the ^32^P-labeled polymerase reaction product. As shown in Figure 5, this product was hydrolyzed in NaOH to yield a species three nucleotides longer than the input 13-mer pDNA_OH_ primer, signifying that the third cycle of primer extension entailed the addition of rCMP opposite the first template G base. In this case, the 5′-labeled alkaline hydrolysis product comprised a mixture of pDNAp(dT)p(dT)p(rC)_2′_p and pDNAp(dT)p(dT)p(rC)_3′_p strands.

To determine whether and how effectively DinB2 can embed a ribonucleotide during DNA synthesis, we presented the enzyme with dATP, dGTP and dTTP (125 μM each) and an equimolar mixture of 63 μM dCTP and 63 μM rCTP. In this case, treatment of the polymerized strand with NaOH resulted in the appearance of three new hydrolysis products (denoted by • in Figure 5) that, from their size, were indicative of rCMP incorporation at the +3, +4 and +13 addition steps, across from guanines in the template strand. By quantifying the radioactivity in the individual alkaline hydrolysis products and in the ensemble of longer extension products, we could calculate the percent ribonucleotide embedded at each C addition step: the values were 17% at the +3 step, 37% at the +4 step and 24% at the +13 step.

### A single amino acid polymorphism governs ribonucleotide utilization

**Figure 6.**
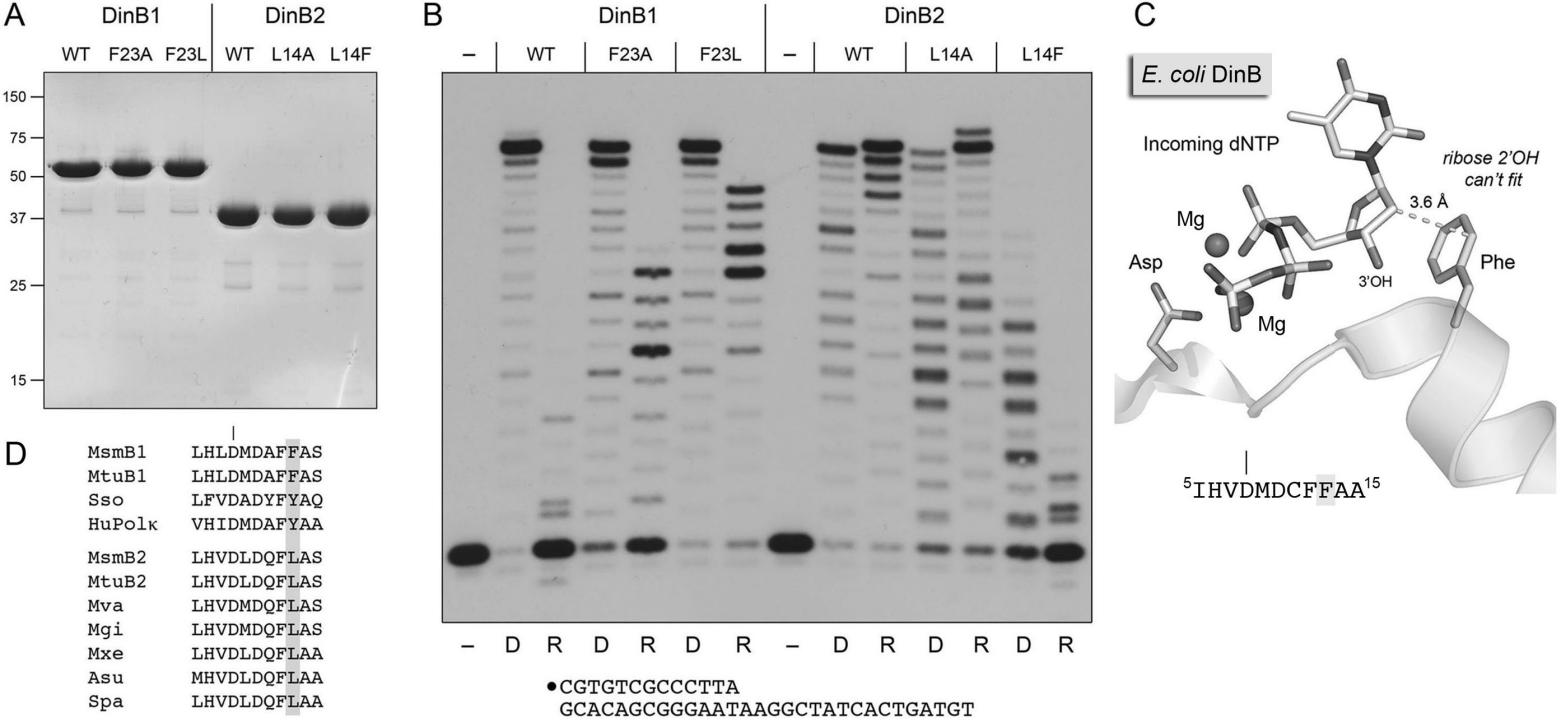
A single amino acid polymorphism governs ribonucleotide utilization. (**A**) Purification. Aliquots (5 μg) of the indicated DinB1 and DinB2 proteins were analyzed by SDS-PAGE. The Coomassie Blue-stained gel is shown. The positions and sizes (kDa) of marker polypeptides are indicated on the left. (**B**) Polymerase reaction mixtures (10 μl) containing 10 mM Tris-HCl, pH 7.5, 5 mM MnCl_2_, 50 nM 5′ ^32^P-labeled 13-mer/30-mer primer–template, dNTPs (125 μM each; lanes D) or rNTPs (125 μM each; lanes R) as specified and 1 μM of the indicated DinB proteins were incubated for 10 min at 37°C. The reaction products were analyzed by urea-PAGE and visualized by autoradiography. (**C**) Structure of *E. coli* DinB in complex with two magnesium ions and an incoming dNTP (from pdb 4IR1). The N-terminal DinB segment containing the first metal-binding aspartate and the steric gate phenylalanine is shown. The amino acid sequence is specified at bottom. (**D**) Distinct clades of DinB homologs with aromatic versus aliphatic steric gates. The amino acid sequences in the vicinity of the metal-binding aspartate and steric gate residues are aligned for exemplary DinB/PolIV homologs that either have an aromatic steric gate side chain (top grouping) or a leucine instead (bottom grouping). The aromatic gate group includes *M. smegmatis* DinB1, *M. tuberculosis* DinB1, *Sulfolobus sulfataricus* Dpo4 and human Pol kappa. The leucine gate clade includes DinB2-like proteins from Actinobacteria species *M. smegmatis*, *M. tuberculosis*, *M. vanbaalenii*, *M. gilvum*, *M. xenopi*, *Amycolicicoccus subflavus* and *Saccharomonospora paurometabolica*.

The ability of DinB2 to efficiently incorporate rNMPs, and to embed ribonucleotides even in the presence of a cognate dNTP, is a distinctive property not shared by DinB1 or DinB3. Inspection of the mycobacterial DinB primary structures revealed that DinB2 has a leucine (Leu14) at the position referred to as the ‘steric gate’, whereas DinB1 and DinB3 have phenylalanine (Phe23) and tyrosine (Tyr20), respectively. The steric gate has been implicated in rNTP discrimination by multiple DNA polymerases (35). In *E. coli* DinB, the steric gate residue Phe13 is situated five amino acids downstream of the metal-binding aspartate Asp8 (Figure 6C) (32). The bulky Phe13 aromatic side chain packs against the deoxyribose sugar of the incoming dNTP, at a distance of 3.6 Å from the C2′ atom, thereby clashing with the ribose 2′-OH group of a potential incoming rNTP (Figure 6C). We hypothesized that the smaller leucine side chain in DinB2, instead of a bulkier aromatic side chain, might open the steric gate to accommodate ribose and thereby account for its ability to utilize rNTPs. To test this idea, we mutated DinB2 Leu14 to phenylalanine and alanine, purified the recombinant L14A and L14F proteins (Figure 6A) and gauged their polymerase activity on the 13-mer/30-mer primer–template with dNTPs and rNTPs as substrates (Figure 6B). The salient finding was that whereas the alanine change did not affect the ability of DinB2 to perform fill-in synthesis with rNTPs, the phenylalanine mutation squelched DinB2's RNA polymerase activity (Figure 6B). The L14F change also diminished DNA polymerase activity relative to wild-type DinB, but to a lesser degree than it affected ribo incorporation. Thus, installing an aromatic steric gate in DinB2 closed the steric gate against ribonucleotides.

In a reciprocal experiment, we queried whether changing DinB1's steric gate Phe23 to leucine or alanine might open its gate and thereby elicit a gain of function in RNA polymerization. We found that the DinB1-F23L mutant acquired vigorous RNA polymerase activity, evinced by its near-quantitative elongation of the input primer, entailing 9–13 steps of rNMP incorporation (Figure 6B). The F23A mutant also gained activity in rNMP incorporation (60% of primers extended; most by 6–9 rNMP addition steps) vis à vis wild-type DinB1 (12% of primers extended), but was not as active in this regard as F23L (Figure 6B). The F23A and F23L changes had no obvious effect on the DNA polymerase activity of DinB1.

We conclude from these experiments that the aromatic–aliphatic polymorphism at the mycobacterial DinB steric gate is a tunable determinant of ribonucleotide utilization. To our knowledge, DinB2 is the first example of a DNA polymerase IV family member that is naturally adept at ribonucleotide incorporation. The studies presented below focus in detail on the biochemical properties of DinB2.

### Kinetics of templated addition of single dNTP and rNTP substrates

**Figure 7.**
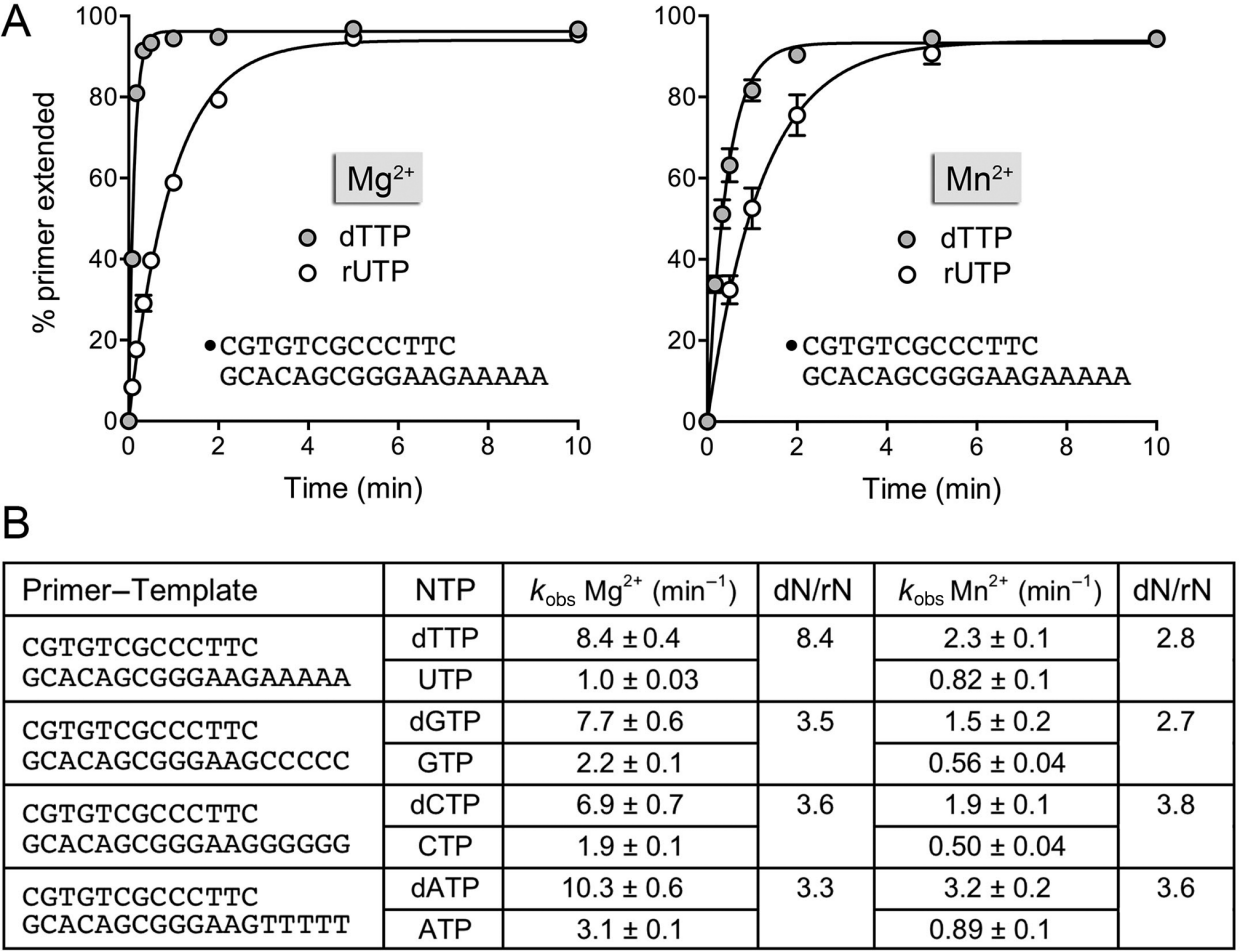
Kinetics of templated dNMP and rNMP incorporation. (**A**) Polymerase reaction mixtures contained 10 mM Tris-HCl, pH 7.5, 50 nM 5′ ^32^P-labeled 13-mer/18-mer primer–template with a homo-oligo-dA tail, and either 5 mM MnCl_2_ and 500 μM dTTP or rUTP (left panel) or 1 mM MnCl_2_ and 100 μM dTTP or rUTP (right panel). Aliquots (10 μl) were withdrawn at the times specified and quenched immediately with EDTA/formamide. The reaction products were analyzed by urea-PAGE and the percent of primer strand extended by one or more nucleotides was quantified by scanning the gel with a Fujix BAS2500 imager. The % primer extension is plotted as a function of reaction time. Each datum in the graphs is the average of three separate experiments ±SEM. The rate constants (*k*_obs_ ± SE) for the first step of dTTP and UTP addition to the primer–template were obtained by non-linear regression curve fitting of the data to a one-phase association function in Prism and are shown in panel (B). A series of 5′ ^32^P-labeled 13-mer/18-mer primer–templates with identical duplex segments and either homo-oligo-dC, homo-oligo-dG or homo-oligo-dT template tails was used to assay the kinetics of G, C and A nucleotide addition to the primer strand, with magnesium or manganese as the metal cofactor. The rate constants are compiled in panel (B). The ratios of the rate of the first deoxynucleotide and ribonucleotide addition steps are indicated in the columns dN/rN.

In order to measure the rates of the first templated nucleotide additions to a DNA primer terminus, we exploited a series of 5′ ^32^P-labeled 13-mer/18-mer primer–templates consisting of an identical 13-bp duplex plus a 5-nucleotide homo-oligomeric 5′ tail, either dA, dC, dG or dT, that would template the addition of one correctly base-paired nucleotide from a dNTP or rNTP substrate. Polymerase reactions were performed in 20-fold DinB2 molar excess over primer–template. Exemplary kinetic data are shown in Figure 7A for the addition of dTMP or rUMP to a primer–template with an oligo-dA tail in the presence of magnesium (left panel) or manganese (right panel) as the divalent cation cofactor. By plotting the percent of primer strand elongated by one or more steps as a function of time, and fitting the data to a one-phase association in Prism, we derived rate constants (*k*_obs_) for the first nucleotide addition step. The full ensemble of rate measurements is compiled in Figure 7B. The following themes emerged: (i) dNMP incorporation was faster than cognate rNMP incorporation in all cases, ranging from 3.3- to 8.4-fold faster with magnesium and 2.7- to 3.8-fold in manganese; and (ii) the rates of nucleotide addition were 3- to 5-fold faster in magnesium than manganese for all dNTPs and rNTPs, with the exception of rUTP. Notably, the rate of dNMP addition in magnesium did not vary significantly (i.e. <2-fold) according to the nucleobase (range 6.9–10.3 min^−1^); the dNMP addition rates in manganese spanned a 2-fold range, from 1.5 to 3.2 min^−1^. DinB2 incorporated rNMPs with rates ranging from 1.0 to 3.1 min^−1^ in magnesium and 0.5 to 0.89 min^−1^ in manganese.

### DinB2 scavenges nucleotides in the presence of manganese

**Figure 8.**
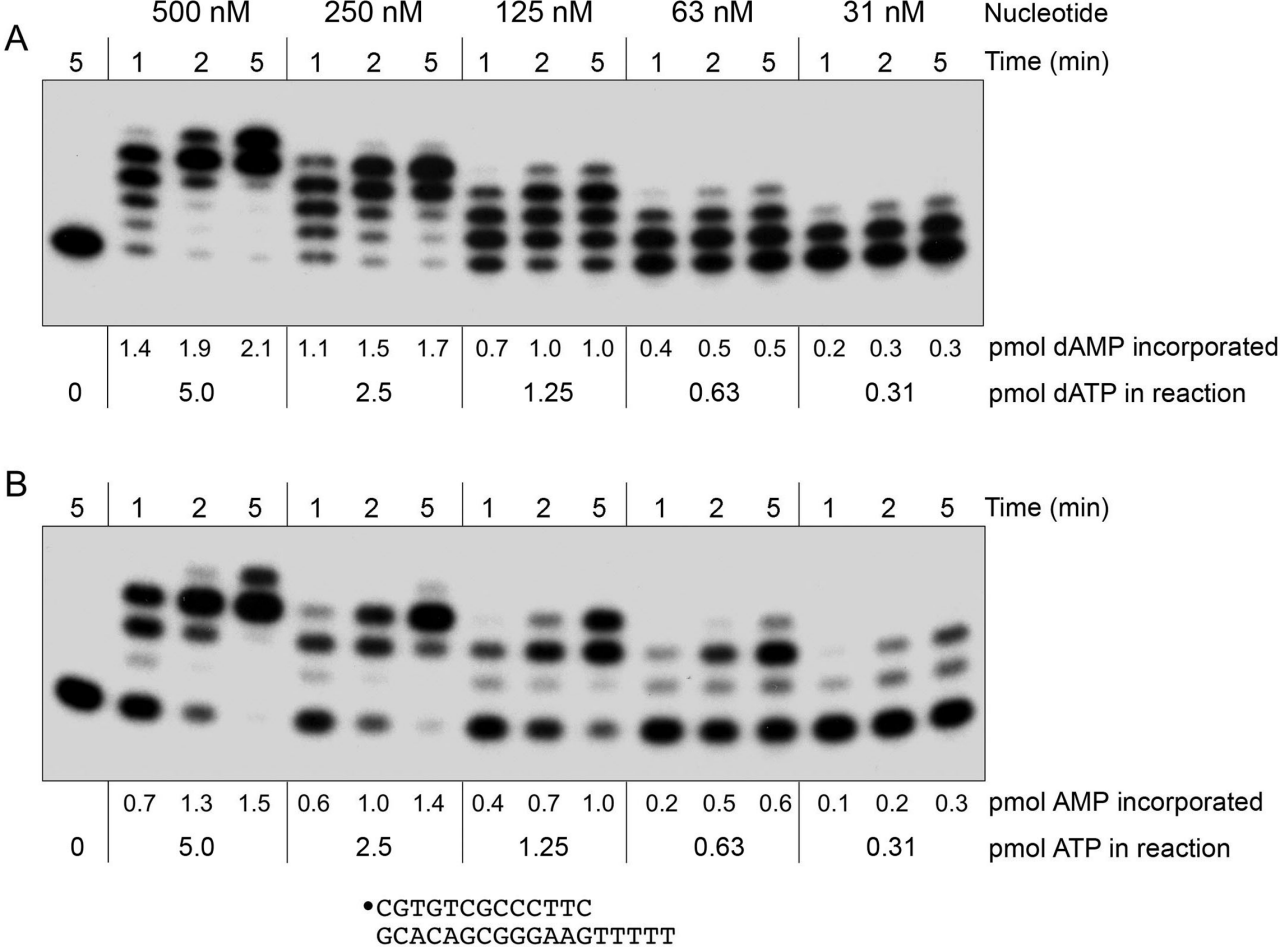
DinB2 scavenges nucleotides in the presence of manganese. Reaction mixtures containing 10 mM Tris-HCl, pH 7.5, 1 mM MnCl_2_, 50 nM 5′ ^32^P-labeled 13-mer/18-mer primer–template with a homo-oligothymidylate tail, 1 μM of DinB2, and either 500 nM, 250 nM, 125 nM, 63 nM or 31 nM dATP (top panel) or ATP (bottom panel) were incubated at 37°C. Aliquots (10 μl) were withdrawn at 1, 2 and 5 min. The reaction products were analyzed by urea-PAGE and visualized by autoradiography. The input amounts (pmol) of dATP or ATP substrates in the 10 μl aliquots of reaction mixture are indicated below the autoradiograms. The gel was scanned with a Fujix BAS2500 imager and the pmol of dAMP/AMP incorporation into the primer strand were calculated as described in the Results section; the values are shown below each lane in the gels.

In an attempt to determine the affinity of DinB2 for cognate dNTP and rNTP in the presence of manganese, we tracked the kinetics of dAMP and AMP addition by 1 μM DinB1 to 50 nM 13-mer/18-mer primer–template–(oligo-dT tail), varying the concentration of the dATP and ATP substrates. We found that when decreasing the nucleotide concentration from 500 μM to 1 μM, there was negligible difference in the rate of primer consumption and the distribution of the extended products (data not shown). We surmised that DinB2 has a high affinity for nucleotides in the presence of manganese. Thus, we back-titrated the dATP and ATP concentrations in serial 2-fold steps to 500, 250, 125, 63 and 31 nM (corresponding to 5.0, 2.5, 1.25, 0.63 and 0.31 pmol of dATP/ATP per 10 μl of reaction mixture) and gauged the primer extension pattern at 1, 2 and 5 min (Figure 8). Because the reaction mixtures contained 50 nM of primer–template with five available template T positions, the upper limit of adenylate additions was 250 nM (which corresponds to 2.5 pmol of adenylates polymerized per 10 μl aliquot of reaction mixture). By quantifying the distribution of the 5′ ^32^P-labeled strands at the +1, +2, +3, +4 and +5 steps, multiplying the concentration of each by the number of nucleotide additions and summing the values, we could gauge how much dAMP/AMP was added and compare this to the amount of dATP/ATP present at the start of the reaction. At 500 nM dATP (a 2-fold excess of nucleotide over available template sites), DinB2 incorporated 2.1 pmol of dAMP at 5 min, filling 84% of the available template while consuming 42% of the available dATP (Figure 8A). At 250 nM dATP (equimolar to template dT), DinB filled 68% of the template sites (consuming 68% of the dATP). At 125 nM dATP, 63 nM dATP and 31 nM dATP, DinB transferred 77%, 83% and 97% of the input nucleotide to the primer terminus respectively (Figure 8A). Similar results were obtained for ATP incorporation (Figure 8B). Thus, DinB2 is extremely effective at scavenging limiting nucleotide substrate in the presence of manganese.

### DinB2 polymerization rates and affinities for individual nucleotide substrates

**Figure 9.**
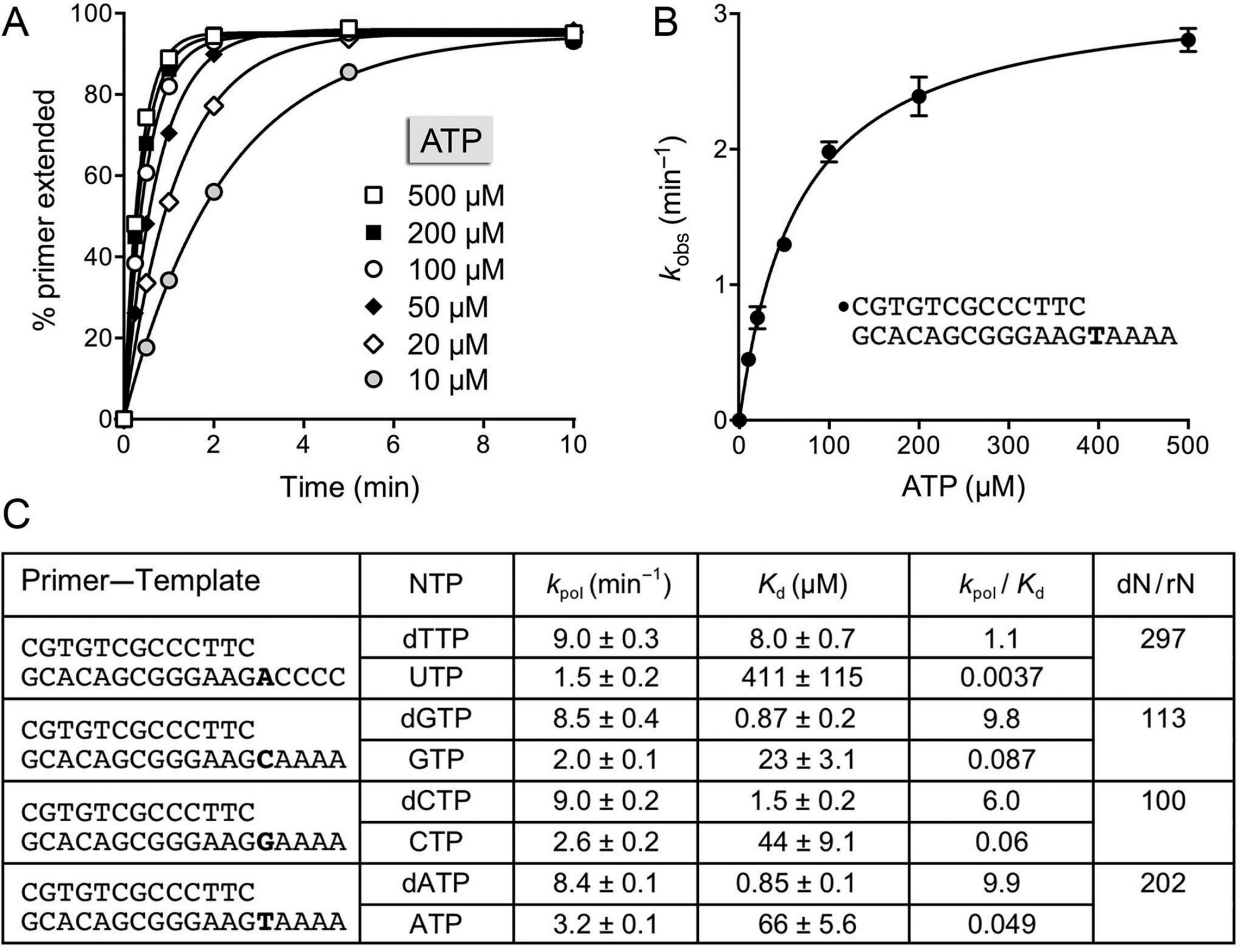
DinB2 polymerization rates and affinities for individual nucleotide substrates with magnesium as metal cofactor. Polymerase reaction mixtures containing 10 mM Tris-HCl, pH 7.5, 5 mM MgCl_2_, 50 nM 5′ ^32^P-labeled 13-mer/18-mer primer–template with dT as the first template nucleobase (shown in panel B), 1 μM DinB2 and the indicated concentrations of ATP were incubated at 37°C. Aliquots (10 μl) were withdrawn at times specified and quenched with EDTA/formamide. The products were analyzed by urea-PAGE and quantified by scanning the gels. (**A**) The % of primer extended is plotted as a function of reaction time for each concentration of ATP substrate. The data were fit by non-linear regression in Prism to a one-phase exponential. (**B**) The *k*_obs_ values calculated in Prism are plotted as a function of ATP concentration; each datum in the graph is the average of two separate experiments. The data were fit by non-linear regression to a single binding function, from which the *K*_d_ for nucleotide and turnover number (*k*_pol_) at saturating nucleotide were derived. (**C**) A series of 5′ ^32^P-labeled 13-mer/18-mer primer–templates with identical duplex segments and either dA, dC, dG or dT as the first template nucleobase was used to assay the kinetics of the indicated nucleotide addition to the primer strand, as a function of dNTP/rNTP nucleotide concentration, with magnesium as the metal cofactor. The data were analyzed as in panels (A) and (B). The *k*_pol_ and *K*_d_ values and *k*_pol_/*K*_d_ ratio (catalytic efficiency) for each dNTP and rNTP substrate are indicated. The ratios of the catalytic efficiencies for deoxynucleotide and ribonucleotide substrates are indicated in the column dN/rN.

In the presence of magnesium, we found that the rate of nucleotide addition displayed a classic dependence on the concentration of the first templated dNTP or rNTP. For this analysis, we used a series of 13-mer/18-mer primer–templates in which the first template nucleobase was varied (as dA, dC, dG or dT) and the next four template nucleobases comprised a homo-oligomer, either dA_4_ (when the first template base was dC, dG or dT) or dC_4_ (when the first template base was dA) (Figure 9B). The purpose here was to track a single nucleotide addition step at the first template position, without the complication of recurrent additions directed by the homo-oligomer sequence. Illustrative kinetic profiles for the addition of ATP to the primer terminus are shown in Figure 9A. The data at each ATP concentration were fit to a one-phase association function; a plot of the apparent rate constants as a function of ATP concentration yielded a hyperbolic curve (Figure 9B) that, upon non-linear regression fitting to a one-site binding site function, yielded an apparent affinity (*K*_d_) of 66 μM ATP and a turnover number (*k*_pol_) of 8 min^−1^. The kinetic parameters for magnesium-dependent templated addition of each dNTP and rNTP substrate are compiled in Figure 9B. The *k*_pol_ values for dNTPs were remarkably similar independent of the nucleobase (range 8.4–9.0 min^−1^) as were the *k*_pol_ values for rNTPs (range 1.5–3.2 min^−1^). Considering just the turnover numbers at saturating nucleotide, DinB2 was between 2.6- and 6-fold faster at incorporating a dNMP than a cognate rNMP.

The salient findings were that *K*_d_ for nucleotide substrate was strongly influenced by the nucleoside sugar and the nucleobase. Among the dNTP series, DinB2 displayed the highest apparent affinity for dATP (0.85 μM) and dGTP (0.87 μM), followed by dCTP (1.5 μM), and dTTP (8.0 μM)—a 9-fold range. Among the rNTPs, DinB2 had the best apparent affinity for GTP (22 μM), then CTP (44 μM) and ATP (66 μM), with UTP finishing a distant last (411 μM)—an 18-fold range. Taking the ratio of *k*_pol_/*K*_d_, as a measure of catalytic efficiency, we see that the ribose sugar has a strong negative effect dominated by the lower affinities for rNTP substrate. We take the ratio of the catalytic efficiencies for cognate dNTP/rNTP substrates as a measure of sugar discrimination with magnesium as the cofactor (Figure 9C); the values ranged from 100 to 297 depending on the nucleobase.

### DinB2 can utilize RNA as a template for DNA synthesis

**Figure 10.**
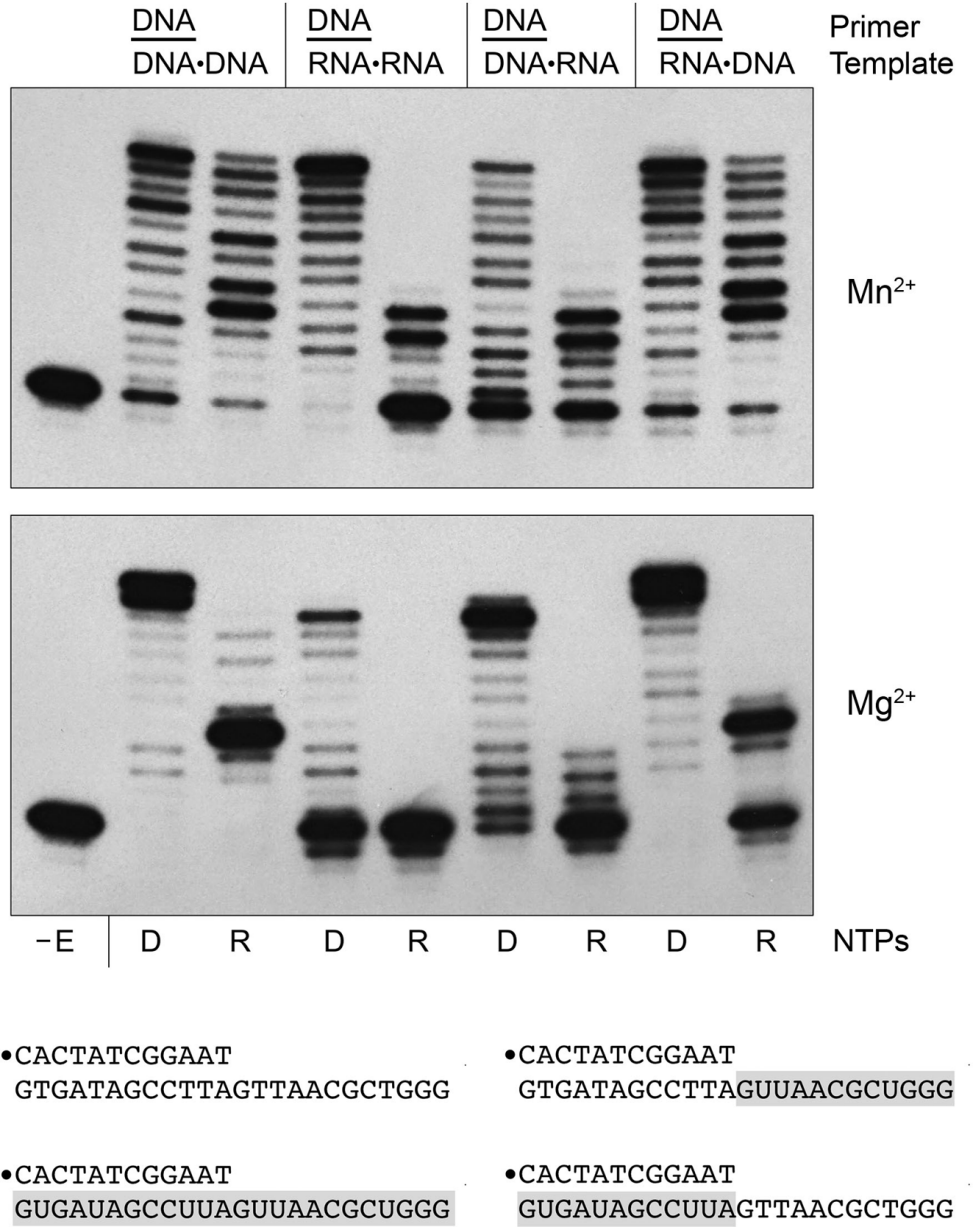
DinB2 can synthesize DNA using an RNA template. Polymerase reaction mixtures (10 μl) containing 10 mM Tris-HCl, pH 7.5, 50 nM 5′ ^32^P-labeled 12-mer/24-mer primer–template as specified, either 1 mM MnCl_2_ and 100 μM of each dNTP or rNTP (top panel) or 5 mM MgCl_2_ and 125 μM of each dNTP or rNTP (bottom panel), and 1 μM DinB2 were incubated at 37°C for 15 min. The reaction products were analyzed by urea-PAGE and visualized by autoradiography. The dNTP (D) or rNTP (R) substrates are indicated below the lanes. DinB was omitted from the control reaction in lane –E. The primer–templates are depicted at bottom with RNA in the template strand shaded gray.

We gauged the ability of DinB2 to incorporate rNMPs and dNMPs instructed by an RNA template. Four 12-mer/24-mer primer–templates were employed, composed of a 12-mer DNA primer strand that paired with a 24-mer template strand that consisted of: (i) all DNA; (ii) all RNA; (iii) a chimera of a 12-nucleotide 3′ RNA segment and a 12-nucleotide 5′ DNA segment, such that the primer duplex is a DNA:RNA hybrid and the template tail is DNA; or (iv) a chimera of a 12-nucleotide 3′ DNA segment and a 12-nucleotide 5′ RNA segment, such that the primer duplex is a DNA:DNA duplex and the template tail is RNA (Figure 10). Primer extension with dNTP or rNTP substrates was assayed in the presence of either 1 mM manganese (Figure 10, top panel) or 5 mM magnesium (Figure 10, bottom panel).

In the presence of manganese and the all-DNA primer–template, DinB2 polymerized dNTPs to generate a ladder of extension products from +1 to +12, of which the +4, +9 and +12 species were the most prominent. On the same template, DinB2 polymerized rNTPs to yield a ladder extending up to +11, featuring prominent species at +4, +5 and +7. The same product distributions were seen when DinB2 reacted with the chimeric primer–template in which the pDNA primer was paired with RNA and the template tail was DNA, suggesting that manganese-dependent DinB2 polymerase activity tolerates a primer strand in an A-form helical conformation. The salient finding was that DinB2 displayed vigorous activity in DNA synthesis on an all-RNA template strand, whereby +12 complete fill-in was the predominant extension product. By contrast, the incorporation of rNTPs was suppressed when the template strand was all-RNA with respect to the fraction of primers elongated and the length of the extension tract (principally to +3 and +4). The pattern of rNMP addition was similar when the duplex primer segment was DNA:DNA and only the template single-strand tail was RNA. We conclude from these and preceding experiments that DinB2 has respectable DNA-dependent RNA polymerase and RNA-dependent DNA polymerase (reverse transcriptase) activities in the presence of manganese, but is relatively feeble as an RNA-dependent RNA polymerase.

In the presence of magnesium, DinB2 polymerized dNMPs similarly on the all-DNA primer–template and the chimeric primer–template with a DNA:RNA duplex and DNA template tail, extending most of the input primer strands to +11 and +12. DinB2 also quantitatively extended the all-DNA primer–template with ribonucleotides, but displayed a strong stop at +4, a point at which the polymerase will have added the first UMP across from an AA dinucleotide in the template. The fraction of primer extended with RNA was lower on the chimeric primer–template with a DNA:RNA duplex and DNA template tail, though the polymerase still stopped after extension to +4. It is conceivable that the +4 stop at AA in the template sequence reflects the comparatively high *K*_d_ of DinB2 for UTP in the presence of magnesium (411 μM; a value greater than the 125 μM UTP included in the RNA polymerase reactions in Figure 10). Magnesium-dependent ribonucleotide addition to the DNA primer was effectively squelched when the template single strand was RNA. DinB2 was adept at adding deoxynucleotides on the RNA template when the primer duplex segment was DNA:DNA, generating a predominant +10 extension product. Reverse transcriptase activity in magnesium was significantly diminished when the template strand was all-RNA. This trend was opposite to what was seen with manganese as the metal cofactor.

## DISCUSSION

This study unveils *M. smegmatis* DinB2 as the founder of a novel clade of DinB polymerase that naturally lacks an aromatic steric gate and is adept at incorporating ribonucleotides with either manganese or magnesium as the metal cofactor. DinB2 efficiently scavenges limiting concentrations of dNTP and rNTP substrates in the presence of manganese, which apparently confers higher affinity for nucleotide substrates than magnesium, as also noted for the human Y-family enzyme DNA polymerase ι (36). DinB2's sugar selectivity factor, determined from the rates of manganese-dependent dNMP versus rNMP incorporation, is 2.7- to 3.8-fold. DinB2 readily embeds ribonucleotides during manganese-dependent DNA synthesis when rCTP is present at the same concentration as dCTP. Moreover, DinB2 can incorporate a tract of up to 16 consecutive ribonucleotides, a feature that distinguishes it from the manganese-dependent RNA polymerase of LigD-POL, which arrests after four steps of ribonucleotide addition (34). In magnesium, DinB2 has a 26- to 78-fold lower affinity for rNTPs than dNTPs. When *k*_pol_/*K*_d_ values are compared for magnesium-dependent deoxy and ribo additions, the sugar selectivity factors are 100–297. Other exemplary Y-family polymerases have sugar selectivity values of 3400–20500 (37,38). Because the concentrations of rNTPs *in vivo* can greatly exceed those of cognate dNTPs (e.g. by 36- to 190-fold in yeast (39)) we suspect that DinB2's sugar discrimination with magnesium under ‘physiological’ circumstances will be dictated by the *k*_pol_ values, which reveal only a 2.6- to 6-fold differential in the rates of deoxy versus ribo addition.

Two other mycobacterial Y-family polymerases, DinB1 and DinB3, are characterized here as template-dependent DNA polymerases that discriminate strongly against ribonucleotide substrates, a property that, in the case of DinB1, we show correlates with its aromatic steric gate side chain. The steric gate of replicative and repair polymerases guards the genome against embedded ribonucleotides by clashing with the 2′-OH of an rNTP substrate. This residue is a glutamate for the A family polymerases, or a phenylalanine or tyrosine for the B- and Y-family polymerases (35). Many studies have verified that subtraction of the aromatic steric gate elicits a gain of function in ribonucleotide utilization (37,38,40,41). We extend this theme here to *M. smegmatis* DinB1 by showing that changing Phe23 to leucine conferred vigorous RNA polymerase activity.

The DinB enzymes characterized previously all have aromatic steric gates. Here we find that the naturally strong RNA polymerase activity of mycobacterial DinB2 is governed by a leucine side chain in lieu of an aromatic steric gate. Is this a one-off property of *M. smegmatis* DinB2? Or might it have broader relevance? We suggest the latter insofar as a phylogenetic analysis reveals that several taxa of the phylum *Actinobacteria* have DinB2-like polymerases that lack the canonical aromatic steric gate residue and instead have a leucine (Figure 6D). These include DinB2 homologs from other species of the *Mycobacterium* genus (e.g. *M. smegmatis*, *M. tuberculosis*, *M. vanbaalenii*, *M. gilvum* and *M. xenopi*) and from two other genera: *Amycolicicoccus subflavus* and *Saccharomonospora paurometabolica*. It will be of interest to test whether these DinB2 enzymes are also naturally adept at polymerizing ribonucleotides. In addition, we identified other steric gate polymorphisms among DinB homologs from *Actinobacteria*, including those with isoleucine (from *Amycolatopsis mediterranei, Kribbella flavida*, *Catenulispora acidiphila*, *Saccharomonospora marina*, *Saccharothrix espanaensi*, *Beutenbergia cavernae*, *Rhodococcus pyridinivorans*, *Kineosphaera limosa* and *Arthrobacter aurescens*) and valine (from *Cellulomonas fimi* and *Thermobispora bispora*).

The distinctive biochemical specificities of the *M. smegmatis* DinB paralogs, taken together with the genetics of *M. tuberculosis* DinB1 and DinB2 (24), prompt speculation regarding their physiological functions. DinB1 is likely involved in lesion bypass during replicative synthesis, aided by its physical association with the β-clamp processivity factor; this interaction is mediated via a DinB1 C-terminal extension that is absent from DinB2 (24) (Figure 1). DinB2 might play a role in gap repair and/or fill-in of 5′ overhangs at DSBs during NHEJ. The lack of an overt phenotype when *dinB1* and *dinB2* are deleted, singly or in combination (24), likely reflects their functional redundancy with other mycobacterial repair polymerases. The RNA polymerase activity of DinB2 resembles, in some regards, the RNA polymerase functions of the mycobacterial AEP enzymes LigD-POL, PolD1 and PolD2, deletions of which also failed to expose an overt phenotype (15). Ultimately, the search for phenotype may require the construction of mycobacterial strains with five or more polymerase gene deletions. Meantime, we remain attracted to the idea that the ability of enzymes such as DinB2 and LigD-like POLs to synthesize a ‘ribo patch’ during DNA repair, especially when dNTPs are limiting (e.g. as is likely during quiescence), is an intelligent strategy for bacterial survival, insofar as the consequences of ribo embedding in DNA are less drastic than death ensuing from an unrepaired break.

With respect to the consequences of ribo embedding, we show here that DinB2 has a reverse transcriptase-like activity that polymerizes DNA on an RNA primer. Thus, DinB2 has not only the capacity to install a ribo patch, it has the added value of being able to replicate across such a patch in the event that the ribonucleotides are not removed before the next round of chromosome duplication.
